# Cell-Free DNA Promotes Inflammation in Patients With Oral Lichen Planus *via* the STING Pathway

**DOI:** 10.3389/fimmu.2022.838109

**Published:** 2022-04-14

**Authors:** Jing Deng, Weiyi Pan, Ning Ji, Na Liu, Qian Chen, Jinhuan Chen, Yutong Sun, Liang Xie, Qianming Chen

**Affiliations:** State Key Laboratory of Oral Diseases, National Clinical Research Center for Oral Diseases, Chinese Academy of Medical Sciences Research Unit of Oral Carcinogenesis and Management, West China Hospital of Stomatology, Sichuan University, Chengdu, China

**Keywords:** oral lichen planus, inflammation, cfDNA, THP-1 macrophages, PAMAM-G3, STING

## Abstract

**Background:**

Damaged and dead cells release cell-free DNA (cfDNA) that activates cyclic GMP–AMP (cGAMP) synthase (cGAS), which leads to the activation of stimulator of interferon genes (STING) *via* the second messenger cGAMP. STING promotes the production of inflammatory cytokines and type I interferons to induce an inflammatory response. Oral lichen planus (OLP), a chronic autoimmune disease involving oral mucosa characterized by the apoptosis of keratinocytes mediated by T-lymphocytes, is related to the activation of multiple inflammatory signaling pathways. Currently, the relationship between cfDNA and OLP has not been confirmed. We hypothesized that cfDNA may be a potential therapeutic target for OLP.

**Methods:**

cfDNA was extracted from the saliva and plasma of OLP patients; its concentration was measured using the Quanti-iT-PicoGree kit and its relationship with OLP inflammation was assessed. cfDNA of OLP patients (cfDNA-OLP) was transfected into THP-1 macrophages and the expression of inflammatory factors was investigated by performing quantitative real time PCR (qRT-PCR), western blotting, and enzyme-linked immunosorbent assay (ELISA). STING expression was analyzed in the tissues of OLP patients and healthy controls using immunohistochemical staining and western blotting. siRNA was used to knockdown *STING* expression in THP-1 macrophages, and the inflammatory cytokines tumor necrosis factor-α (TNF-α) and interleukin-6 (IL-6) secreted by cells following cfDNA-OLP transfection were detected using ELISA. Finally, the effect of the cationic polymer PAMAM-G3 was evaluated on the treatment of inflammation induced by cfDNA-OLP.

**Results:**

The concentration of cfDNA in the saliva and plasma of OLP patients was considerably higher than that of healthy controls, and it positively correlated with the levels of inflammatory cytokines and clinical characteristics. cfDNA-OLP induced an inflammatory response in THP-1 macrophages. STING expression was significantly higher in OLP tissues than in the gingival tissues of healthy controls. STING knockdown suppressed cfDNA-OLP-induced inflammation in THP-1 macrophages. PAMAM-G3 inhibited the inflammatory response caused by cfDNA-OLP.

**Conclusion:**

The cfDNA level is increased in OLP patients, and the STING pathway activated by cfDNA-OLP might play a critical role in OLP pathogenesis. Treatment with PAMAM-G3 reduced the inflammation induced by cfDNA-OLP, and therefore, may be a potential treatment strategy for OLP.

## Introduction

Global incidence of oral lichen planus (OLP), a T-lymphocytes-mediated chronic autoimmune disease, is 1.01% and particularly common in middle-aged individuals and women (1). Currently, OLP is considered a potentially malignant disorder with cancer developing in approximately 1% of afflicted patients, although this estimate is debatable (2, 3). Pathologically, OLP manifests as dense subepithelial lymphocyte infiltration accompanied with degenerating intraepithelial lymphocytes and basal keratinocytes (4). The mechanisms involved in OLP include antigen presentation; activation, proliferation, and migration of lymphocytes; and apoptosis of keratinocytes caused by CD8^+^ cytotoxic T-cells. During OLP progression, multiple cells including keratinocytes, Langerhans cells, CD4^+^/CD8^+^ T lymphocytes, macrophages, and mast cells secrete cytokines and chemokines to promote inflammation (5). Antigen presentation by basal keratinocytes is considered the key step causing accumulation of T-cells in the superficial lamina propria, disruption of basement membrane, and apoptosis of keratinocytes caused by CD8+ cytotoxic cells (6). However, the specific antigen of OLP remains unknown. Current treatment strategies for OLP primarily involve anti-inflammatory drugs, immunosuppressive agents, steroids, and biological agents (7). However, the treatment options of OLP have several limitations, including drug resistance, adverse effects, and a high risk of relapse (8). Thus, an improved understanding of the mechanisms underlying OLP is needed to develop novel therapeutic strategies for this disease.

Cell-free DNA (cfDNA), a double-stranded DNA similar to apoptotic DNA, is characterized by high fragmentation and low molecular weight (9). Apoptotic or dead cells, erythroid precursors, mitochondria, and neutrophil extracellular traps (NETs) release DNA fragments, which are degraded to cfDNA (10, 11). cfDNA has been reported to be associated with RA, SLE, and other autoimmune diseases (12, 13). Higher plasma cfDNA levels are related to an increased rate of deterioration, whereas lower cfDNA levels correspond to an improvement in clinical symptoms (14). cfDNA can be recognized by DNA sensors to activate cell signaling systems, generating a series of pro-inflammatory cytokines to activate the adaptive immune response (15). To date, various DNA sensors recognizing cfDNA have been discovered, including TLR9 on endosomes, and AIM2 and cGAS in the cytoplasm (15). cfDNA activates TLR9, thereby upregulating the expression of nuclear factor kappa B (NF-κB) and interferon regulatory factor 7 (IRF7) (16). NF-κB is involved in the upregulated expression of cytokine genes that promote inflammation, whereas IRF7 upregulates the expression of type I interferons (IFN-α and IFN-β) (17). Activated AIM2 promotes the formation of AIM2 inflammasomes, followed by the activation of caspase-1, maturation and secretion of interleukin (IL)-1β and IL-18, and eventual induction of pyroptosis (18). Upon the direct binding of cfDNA, cGAS catalyzes the synthesis of the cyclic dinucleotide 2’-3’-cGAMP that activates STING, promoting the production of type I interferons *via* IRF3 and that of several pro-inflammatory cytokines (e.g., IL-6 and TNF-α) *via* NF-κB (19, 20). Thus, cfDNA has the potential to reveal the unknown antigens of OLP and the pathogenic mechanisms underlying OLP as a chronic autoimmune disease.

In general, critical roles of cfDNA in inflammation and autoimmunity have been reported, however, to date no study has explored the correlation between cfDNA and OLP. Here, we hypothesized that T cells induced basal keratinocyte apoptosis, resulting in many cfDNA fragments abnormally accumulating in OLP lesions, further promoted the inflammation in OLP *via* activating multiple signaling pathways such as TLRs/NF-κB and cGAS-STING. To establish evidence supporting the pathological role of cfDNA in OLP, we analyzed the cfDNA isolated from the saliva and plasma of 34 OLP patients and 20 healthy subjects in this study. Our findings identify the inflammatory properties of cfDNA-OLP and provide a potential therapeutic strategy to reduce cfDNA-induced inflammation in OLP.

## Materials and Methods

### Participants

All OLP patients and healthy subjects were recruited from the West China Hospital of Stomatology, Sichuan University. The criteria for the inclusion of patients and identification of OLP were based on the modified diagnostic criteria of the World Health Organization (21), OLP patients were diagnosed based on clinical manifestations and biopsy specimen (4), patients over 18 years without skin involvement were included. Exclusion criteria including: patients with lichenoid lesions, suspected lichenoid, and other serious oral mucosal diseases; patients with uncontrolled hypertension or diabetes, tumors, and immune system diseases such as Sjögren’s syndrome and rheumatoid arthritis; pregnancy or breastfeeding women; OLP patients undergoing treatment; patients using corticosteroids or immunomodulators within 3 months. The Institutional Ethical Committee of West China Hospital of Stomatology, Sichuan University approved this study, written consent was obtained from each participants before starting the study.

Saliva and plasma samples were obtained from 34 OLP patients and 20 healthy subjects without any clinically visible buccal inflammation. OLP tissue samples from three OLP patients and healthy oral mucosa tissues from three volunteers who underwent wisdom tooth extraction were also obtained. The clinical information of OLP patients recruited in this study is provided in **Table 1**.

**Table 1:** Clinical parameters of OLP patients recruited in this study.

No.	Sex	Age	Site	Erosion
1	Female	49	Tongue	No
2	Female	66	Buccal	No
3	Female	38	Lips	No
4	Female	70	Buccal	No
5	Male	38	Buccal	No
6	Male	47	Tongue	No
7	Male	32	Tongue	No
8	Female	43	Buccal	No
9	Male	55	Buccal	No
10	Female	46	Tongue	No
11	Female	59	Buccal	No
12	Female	71	Buccal	No
13	Female	49	Buccal	No
14	Female	52	Tongue	No
15	Male	23	Tongue	No
16	Female	38	Buccal	No
17	Female	66	Buccal	No
18	Male	39	Lips	No
19	Female	38	Buccal	No
20	Male	33	Tongue	No
21	Female	47	Buccal	Yes
22	Female	49	Tongue	Yes
23	Male	43	Buccal	Yes
24	Female	76	Buccal	Yes
25	Female	54	Buccal	Yes
26	Female	68	Buccal	Yes
27	Female	62	Tongue	Yes
28	Male	61	Buccal	Yes
29	Male	30	Buccal	Yes
30	Female	55	Buccal	Yes
31	Male	73	Buccal	Yes
32	Male	74	Buccal	Yes
33	Male	63	Buccal	Yes
34	Male	69	Tongue	Yes

### cfDNA Extraction and Quantification

To extract cfDNA from OLP patients and healthy subjects, 3–5 mL of fasting blood sample was collected in EDTA-containing tubes. Then, the samples were centrifuged at 3,000 rpm for 15 min at 4°C, plasma was aspirated, centrifuged at 15,000 rpm for 10 minutes at 4°C and the supernatant was collected. Similarly, 2 mL of non-irritating saliva was collected and eating, drinking, or taking any oral hygiene measures were prohibited within 2 h of collecting the sample. These samples were then centrifuged at 3,000 rpm for 15 min at 4°C, the supernatant was aspirated, then, centrifuged at 15,000 rpm for 10 minutes at 4°C and finally the supernatant was collected. All samples were stored at −80°C until further analysis.

The QIAamp DNA Blood Kit (Cat. #51104; Qiagen) was used to extract cfDNA from the saliva, plasma, and culture supernatants. The concentration of cfDNA was determined using the Quant-iT™ PicoGreen™ dsDNA Assay Kit (Cat. #P11496, Invitrogen) according to the manufacturer’s instructions.

### Cell Culture

Dr. Chen Qianming (State Key Laboratory of Oral Diseases, Sichuan University, China) provided the human oral keratinocytes (HOKs) cell line. The HOKs were cultured in Keratinocyte Serum-Free Medium containing penicillin/streptomycin (100 U/mL; Gibco) at 37°C under 5% CO_2_. The medium was replaced with fresh medium after one day, and the cells were incubated again until they reached 50% confluence. We used 0.05% trypsin-EDTA (Sigma-Aldrich) to digest the cells. The cells were incubated with lipopolysaccharide (LPS; Sigma-Aldrich) for 24 h to mimic the OLP condition *in vitro*. The human monocytic THP-1 cells were purchased from ATCC and cultured (1 × 10^6^ cells/mL) in RPMI 1640 medium (Gibco) with 10% fetal bovine serum (Gibco) and penicillin/streptomycin (100 U/mL) contained at 37°C under 5% CO2. When the cell density reached 2–5 × 10^6^/mL, the medium was replaced with the complete medium containing Phorbol-12-myristate-13-acetate (PMA; 100 ng/mL; Sigma-Aldrich). After 24 h PMA stimulation, THP-1 monocytes differentiated into THP-1 macrophages, after which, the cells were further cultured for two days. All experiments were repeated three times.

### Internalized cfDNA to Stimulate Cells

To evaluate the effect of cfDNA in activating immunity response, the cfDNA extracted from 1 mL mixed saliva or plasma of respectively five OLP patients or five healthy subjects was transfected into THP-1 macrophages (5 × 10^5^ cells/well seeded in 24-well plates) with Lipofectamine 3000 (Lipo3000; Invitrogen) following the manufacturer’s protocol. After 24 h, qRT-PCR, WB and ELISA were conducted to evaluate the pro-inflammation capability of cfDNA in THP-1 macrophages. Twenty-four hours later, the same procedure was performed to stimulate HOKs (5 × 10^5^ cells/well seeded in 24-well plates) with cfDNA and the levels of pro-inflammation cytokines in HOK were evaluated using ELISA. Wells with an equal volume of Lipo3000 were used as controls.

To compare the pro-inflammation capability of cfDNA-OLP to cfDNA-Health and cfDNA-plasma to cfDNA-saliva at the same concentration, 200ng of cfDNA-OLP, cfDNA-Health and 100ng of cfDNA-plasma and cfDNA-saliva was internalized to stimulated THP-1 macrophages for 24 h. The levels of TNF-α and IL-6 in culture supernatants were evaluated *via* ELISA.

### Quantitative Real-Time Polymerase Chain Reaction

TRIzol reagent (Invitrogen) was used to isolate the total RNA for qRT-PCR according to manufacturer instructions. PrimeScript RT Master Mix (TaKaRa Biomed) was used to synthesize the cDNA from isolated RNA. The QuantStudio5 Real-Time PCR system (Applied Biosystems) was used to perform qRT-PCR. The relative amount of mRNA transcripts was calculated using the 2^-ΔΔCt^ formula. *GADPH* was used as the internal control. The PCR primers are provided in **Supplemental Table 1**.

### Western Blotting

RIPA buffer (Beyotime) was used to lyse the cells and tissue samples and obtain total protein. The protein samples from each group were separated by 12% sodium dodecyl sulphate–polyacrylamide gel electrophoresis, next, the resolved proteins were transferred onto polyvinylidene difluoride membranes. Bovine serum albumin (BSA, 5%) was used to block the membranes at room temperature for 1 h, and then incubated with specific primary antibodies overnight at 4°C. The primary antibodies specific to the following proteins and their dilutions were as follows: α-tubulin (1:2000; Cell Signaling Technology); TNF-α, IL-6, p-STING and STING (1:1000; Cell Signaling Technology). The membranes were incubated with relevant secondary antibodies (1:3000; ZSGB-BIO) at 37°C for 1 h. Enhanced chemiluminescence western blotting detection reagent (Millipore) was used to visualize the protein bands. The quantitative data represent the relative ratio of the target protein to total α-tubulin or STING.

### ELISA

The concentration of TNF-α and IL-6 in the saliva, plasma, and culture supernatants was determined using the QuantiCyto^®^ Human ELISA kit (EHC007.96, EHC103a.96; NeoBioscience) following the manufacturer’s protocol. Varioskan LUX microplate reader (Thermo Scientific) was used to measure the absorbance of the samples at 450 nm.

### Immunostaining

The OLP and healthy tissue samples were fixed with 10% formalin, embedded in paraffin, and cut into 5-μm thick sections. For immunostaining, the sections were treated with anti-STING antibody (1:500; Cell Signaling Technology) overnight at 4°C, followed by incubation with secondary antibody (PV-9001; ZSGB-BIO) and the DAB Horseradish Peroxidase Color Development Kit (P0202, Beyotime) in accordance with manufacturer’s instructions. Images were acquired using the Aperio ScanScope scanning microscopic imaging system (Aperio).

### siRNA Knockdown Assay

siRNAs to knock down *STING* were purchased from RIBOBIO (China, Guangzhou). siRNA (200 nM) was transfected into THP-1 macrophages using Lipo3000 following the manufacturer’s protocol. After transfection for 48 h, THP-1 macrophages were transfected with cfDNA-OLP using Lipo3000 for 24 h. Nonspecific siRNA was used as a negative control. The siRNA sequences used in this study are listed in **Supplemental Table 2**.

### Blocking Internalized cfDNA-Mediated Cell Stimulation

To assess the ability of PAMAM-G3 (Sigma-Aldrich) to inhibit the stimulation caused by internalized cfDNA, cfDNA extracted from a 1 mL saliva sample obtained from OLP patients and healthy controls was transfected into THP-1 macrophages (5 × 10^5^/well seeded in 24-well plates) for 4 h. Following this, the medium was replaced with fresh medium containing PAMAM-G3 (25 μg/mL). After a 24 h incubation period, the concentration of TNF-α and IL-6 in supernatants of THP-1 cell was measured using ELISA. The cells were also collected to evaluate the expression of TNF-α, IL-6, and STING using western blotting.

### Cell Viability Assay

Cell viability was assayed using a Cell Counting Kit-8 (CCK-8; Invitrogen). Briefly, THP-1 macrophages (1.0 × 10^4^ cells/well) were seeded in 96-well microplates and cultured for 24 h. The cells were then incubated with PAMAM-G3 at different concentrations (0, 5, 10, 25, 50, 100, and 200 mg/mL) for 24 h. Thereafter, the medium was replaced with RPMI 1640 medium (Gibco) containing 10% CCK-8 reagent and cells were incubated at 37°C for 1 h. The absorbance was measured using a Varioskan LUX microplate reader (Thermo Scientific) at 450 nm.

### Statistical Analysis

Prism 8.0 software (GraphPad) was used to perform a Student’s *t-*test and one-way analysis of variance (ANOVA). Results with *p*< 0.05 were considered statistically significant. All data are expressed as mean ± standard error of the mean (SEM) and are representative of at least three independent experiments. Image J software was used to quantify western blotting results.

## Results

### Increased cfDNA Level Was Associated With Inflammation in OLP Patients

**Figure 1:**
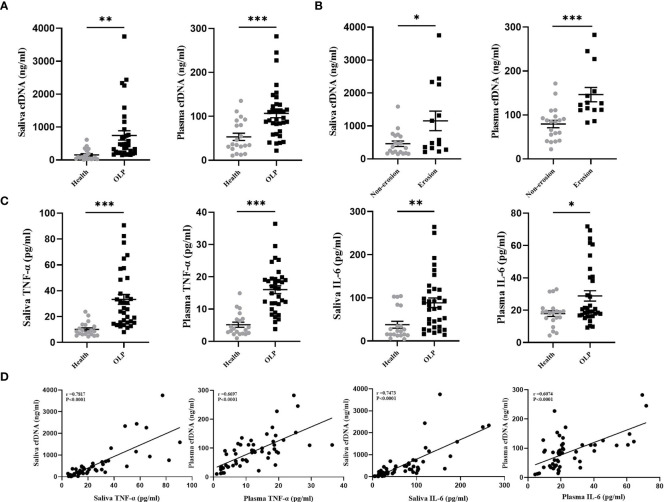
Increased cfDNA Level was Associated with Inflammation in OLP patients. **(A)** cfDNA levels in the saliva and plasma collected from OLP patients (n=34) and healthy subjects (n=20) were measured by Quant-iT PicoGreen dsDNA Assay Kit. **(B)** cfDNA levels in the saliva and plasma collected from patients with non-erosive OLP lesions (n=20) and patients with erosive OLP lesions (n=14) were measured by Quant-iT PicoGreen dsDNA Assay Kit. **(C)** Levels of TNF-α and IL-6 in saliva and plasma of OLP patients (n=34) and healthy subjects (n=20) were determined by ELISA. **(D)** Correlation of fold-change between TNF-α, IL-6, and cfDNA in saliva and plasma from OLP patients (n=34) (Spearman’s correlation test for cfDNA). The data are mean ± SEM (*P<0.05, **P<0.01, ***P<0.001).

cfDNA activates DNA sensors to upregulate the expression of NF-κB, IRF7, IL-6, IL-1β, IL-18, TNF-α, and IFN-α (22–24), those cytokines are associated with the immunopathogenesis of OLP (25). In this study, the concentrations of cfDNA extracted from the saliva and plasma of OLP patients (n= 34) and healthy subjects (n= 20) were evaluated using Picogreen-based assay in order to clarify the relationship between cfDNA and inflammation in OLP patients. The data suggested that OLP patients showed a high level of cfDNA in the saliva compared with the healthy controls (770.1 ± 160.4 vs. 153.8 ± 36.8 ng/mL, *P* = 0.0029) (**Figure 1A**). Similarly, the levels of cfDNA in plasma samples of OLP patients were significantly higher than the samples from the healthy controls (104.8 ± 9.2 vs. 53.3 ± 8.3 ng/mL, *P* = 0.0004) (**Figure 1A**). These data confirmed that there were substantially increased cfDNA levels in plasma of OLP patients, and the extremely high concentration of cfDNA in saliva strengthens the suggestion that cfDNA may be implicated in both the onset and development of OLP. Consistent with our results, patients with Sjogren’s syndrome or SLE also exhibited high levels of cfDNA in plasma (13, 26).

Patients with erosive OLP were reported to correlated with severe inflammation and cell destruction (27, 28), thus, to further define the clinical relevance of cfDNA-OLP, we investigated the association between cfDNA levels and erosion in OLP patients. OLP patients were divided into the erosive group (n = 14) and non-erosive group (n = 20). As expected, patients with erosive lesions showed higher levels of cfDNA than patients without erosive lesions in the saliva (1127 ± 278.8 vs. 387.5 ± 57.2 ng/mL, *P* = 0.0182) (**Figure 1B**) or in the plasma (148.4 ± 13.5 vs. 74.9 ± 6.9 ng/mL, *P* < 0.0001) (**Figure 1B**). TNF-α and IL-6, as pro-inflammatory cytokines, were reported to contribute to the pathogenesis of OLP (29, 30). In this study, we determined that the levels of TNF-α and IL-6 in the saliva and plasma of OLP patients were higher than those in the healthy controls (**Figure 1C**), moreover, the levels of cfDNA in OLP patients were positively correlated with the levels of TNF-α and IL-6 (**Figure 1D**). To be additional, HOKs were treated with LPS for 24 h to mimic the conditions of infection-induced OLP *in vitro*, then significantly increased levels of cfDNA were observed in supernatants compared to that in controls (**Supplementary Figure 1A**), and were also accompanied with higher levels of TNF-α and IL-6 expression (**Supplementary Figure 1B**). These results provided the first evidence that the increased levels of cfDNA contributed to the inflammation in OLP.

### Internalized cfDNA-OLP Induced Inflammation

**Figure 2:**
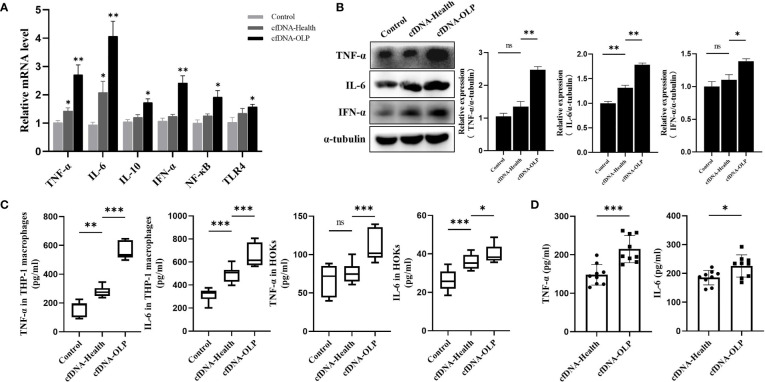
Internalized cfDNA-OLP Induced Inflammation. **(A)** The expression of inflammatory genes in THP-1 macrophages was measured by qPCR. THP-1 macrophages were treated with cfDNA-OLP and cfDNA-Health for 24 h. **(B)** Western blotting was performed to quantify the expression of TNF-α, IL-6, and IFN-α in THP-1 macrophages after 24 h of incubation with cfDNA-OLP and cfDNA-Health. **(C)** THP-1 macrophages and HOKs were treated with cfDNA-OLP and cfDNA-Health for 24 h, the levels of TNF-α and IL-6 in the supernatants were detected by ELISA. **(D)** THP-1 macrophages were treated with cfDNA-OLP and cfDNA-Health at same doses (200 ng/5 × 10^5^ cells) for 24 h, the levels of TNF-α and IL-6 in the supernatants were detected by ELISA. The data are mean ± SEM (*P<0.05, **P<0.01, ***P<0.001). ns, not significant.

To evaluate the pro-inflammation capability of cfDNA-OLP and cfDNA-Health, cfDNA isolated from saliva of OLP patients and healthy subjects was used to stimulate THP-1 macrophages differentiated from THP-1 cells inducted by PMA. In order to avoid interference of the high variation in cfDNA concentrations in OLP patients, we mixed saliva/plasma samples from five separate OLP patients or healthy subjects to obtain enough cfDNA to stimulate inflammatory cells. Results showed that cfDNA-OLP significantly upregulated the expression of pro-inflammatory cytokine genes, especially *TNF-α*, *IL-6*, and *IFN-α* (**Figure 2A**), consistent results were also obtained from western blotting (**Figure 2B**). Finally, the ELISA assay demonstrated that cfDNA-OLP stimulated THP-1 macrophages to produce a large number of inflammatory cytokines (TNF-α, IL-6) (**Figure 2C**). In addition to the THP-1 macrophages, cfDNA-OLP also induced significant inflammatory response in HOKs (**Figure 2C**). Notably, although both the cfDNA-OLP and cfDNA-Health induced inflammation, cfDNA-OLP possessed a more powerful effect on promoting inflammation than cfDNA-Health. cfDNA-OLP in plasma also induced higher levels of TNF-α than cfDNA-Health, but no difference in levels of IL-6 (**Supplementary Figure 2B**). Next, we investigated the pro-inflammation capability of cfDNA-OLP and cfDNA-Health at the same dose (200 ng/5 × 10^5^ cells), and observed that cfDNA-OLP still induced significant inflammatory response in THP-1 macrophages, even once diluted (**Figure 2D**). The pro-inflammation capability of cfDNA-saliva and cfDNA-plasma at the same dose (100 ng/5 × 10^5^ cells) was also investigated, cfDNA-saliva showed higher pro-inflammation capability than cfDNA-plasma (**Supplementary Figure 2C**). These results suggest that the high concentration and specific DNA sequences contributed to the high pro-inflammation capability of cfDNA-OLP.

### cfDNA-OLP Promoted Inflammation by Activating STING

**Figure 3:**
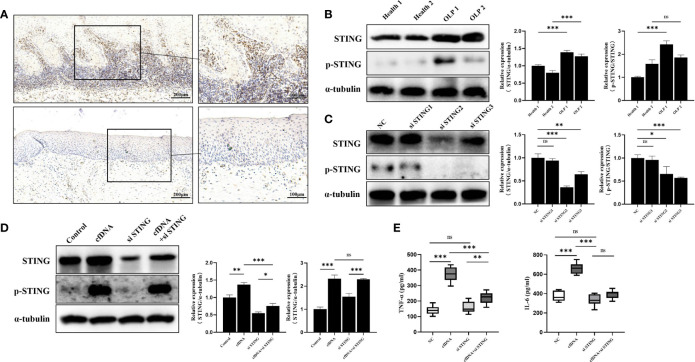
cfDNA-OLP Promoted Inflammation by Activating STING. **(A)** STING expression in OLP tissues was visualized by immunohistochemistry (IHC) staining. Scale bar: 200 and 100 μm. **(B)** The expression of STING in healthy tissues (n=2) and OLP tissues (n=2) was assessed by western blotting. **(C)** THP-1 macrophages were transfected with anti-STING siRNA for 48 h. The protein levels of STING and p-STING was detected by western blotting. **(D)** Western blotting was performed to measure the protein levels of STING and p-STING in STING-knockdown THP-1 macrophages incubated with cfDNA-OLP for 24 h. **(E)** ELISA was performed to measure the levels of TNF-α and IL-6 in the supernatants of STING-knockdown THP-1 macrophages treated with cfDNA-OLP for 24 h. The data are mean ± SEM (*P<0.05, **P<0.01, ***P<0.001). ns, not significant.

STING, a crucial DNA sensor in immune system, were previously shown to be activated to strongly promote inflammatory response (31), and TNF-α and IL-6 were the downstream molecules of the cGAS-STING pathway (32). Thus, we hypothesized that cfDNA promoted inflammation by activating STING in OLP. To test this, we performed western blotting and immunohistochemistry to detect the expression and distribution of STING in OLP and healthy tissues. The immunohistochemical analysis revealed that the number of stained cells in the OLP tissues was significantly higher than in the healthy controls (**Figure 3A**). Interestingly, STING was mainly expressed in the band-like zone of cellular infiltration (containing mainly lymphocytes) rather than keratinocytes. To confirm the immunohistochemical results, we further performed western blotting whose results showed that the expression of STING in OLP tissues was significantly higher than in healthy controls (**Figure 3B**). STING phosphorylation at Ser366 is required for activation in the DNA-sensing pathway (33), hence, we evaluated the protein levels of phosphoryl-STING (p-STING). Western blotting results showed that the levels of p-STING in OLP tissues were much higher than in healthy controls (**Figure 3B**).

To further clarify the mechanism by which cfDNA contributed to inflammation in OLP, three siRNAs were designed to knock down STING in THP-1 macrophages, and western blotting results showed that the protein levels of the STING and p-STING were significantly decreased by siRNAs (**Figure 3C**), among which, siRNA-STING2 that showed the best inhibitory effects was selected for the subsequent experiments. The western blotting results suggested that cfDNA-OLP increased the protein levels and phosphorylation of STING (**Figure 3D**), whereas the increased levels of STING and p-STING were reversed by siRNA-STING. Consistent with the western blotting results, the increased levels of TNF-α and IL-6 induced by cfDNA-OLP was also reversed by STING knockdown (**Figure 3E**). Taken together, our results suggested that cfDNAs in OLP promoted inflammation by activating the phosphorylation of STING.

### PAMAM-G3 Neutralized Inflammation Induced by cfDNA-OLP

Irrespective of excessive production or inadequate clearance of cfDNA, cfDNA deposition is likely to be pathological, because cfDNA can act as an autoantigen to induce autoimmunity and promote inflammation (34). cfDNA is difficult to neutralize with complementary sequence or structure specificity (35), however, cationic polymers were reported to be used for treatment of RA (36), severe sepsis (37) and flu infection (38) by removing cfDNA (39, 40). Thus, removing cfDNA offers a promising potential strategy to treat OLP patients.

**Figure 4:**
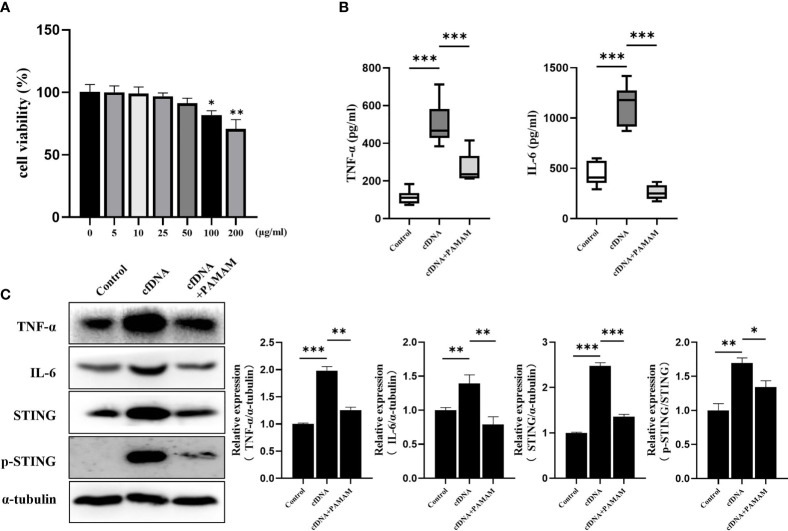
PAMAM-G3 Neutralized Inflammation Induced by cfDNA-OLP. **(A)** Viability of THP-1 macrophages incubated with PAMAM-G3 at concentrations of 0–200 mg/ml for 24 h was measured using CCK-8. **(B)** After treatment with cfDNA-OLP for 4 h, THP-1 macrophages were incubated with PAMAM-G3 (25 μg/ml) for 24 h, and the levels of TNF-α and IL-6 in the supernatants of THP-1 macrophages were determined by ELISA. **(C)** The protein levels of TNF-α, IL-6, STING and p-STING were measured by western blotting. The data are mean ± SEM (*P<0.05, **P<0.01, ***P<0.001).

PAMAM-G3, one of the nucleic acid-binding polymers (NABP), was reported to neutralize the effects of cfDNA (41). After incubation with cfDNA-OLP for 4 h and PAMAM-G3 for 24 h, the levels of TNF-α, IL-6, STING and p-STING in THP-1 macrophages were evaluated. PAMAM-G3 reversed the increased protein levels of TNF-α, IL-6 and STING induced by cfDNA-OLP (**Figure 4C**), in addition, the phosphorylation of STING was also decreased. Consistently, ELISA results showed that PAMAM-G3 inhibited the production of pro-inflammatory cytokines induced by cfDNA-OLP (**Figure 4B**). However, PAMAM-G3 alone did not induce significant inflammation in THP-1 macrophages (**Supplementary Figure 2A**). Although PAMAM molecules reportedly elicit toxicity *in vitro* and *in vivo* owing to the high positive charge of the G3-G6 compounds (42, 43), we found that the viability of THP-1 macrophages was significantly inhibited upon treatment with PAMAM at 100 μg/mL and above; however, no significant inhibition of cell viability was observed when treated with 25 μg/mL PAMAM-G3 (**Figure 4A**).

## Discussion

To the best of our knowledge, this is the first study to report an association between cfDNA and OLP, which contributes to clarify the pathogenic mechanisms of OLP. Our results demonstrated that the cfDNA levels in the saliva and plasma of OLP patients was significantly higher than in the healthy individuals, and there were higher levels of cfDNA in the saliva than in the plasma. Inflammation is always accompanied by massive apoptosis, necrosis and NETosis that are the source of a large amount of cfDNA (44), which explains the increased cfDNA in saliva and plasma of OLP patients. Supporting this view was that higher cfDNA levels are also associated with erosive OLP and higher levels of cytokines, suggesting that the elevated levels of cfDNA were caused by the inflammation of OLP. Consistent with our findings, increased cfDNA levels in the plasma was reportedly associated with inflammation in patients with SLE and celiac disease (13, 45), cfDNA was considered as a biomarker of inflammatory diseases. To evaluate the pro-inflammatory potential of cfDNA, we internalized cfDNA-OLP to induce inflammation in THP-1 macrophages and HOKs, it was noted that cfDNA-OLP significantly upregulated the expression of inflammation-related genes and increased the levels of pro-inflammatory cytokines in THP-1 macrophages, the similar results were observed in HOKs, which were consistent with the results of Liang et al. (46), thus the increased cfDNA in OLP promoted the induction of pro-inflammatory responses. In addition to high concentration, the cfDNA-OLP exhibited higher pro-inflammation capability than cfDNA-Health, even in same concentration, suggesting that the high concentration and specific DNA sequences contributed to the high pro-inflammation capability of cfDNA-OLP.

**Figure 5:**
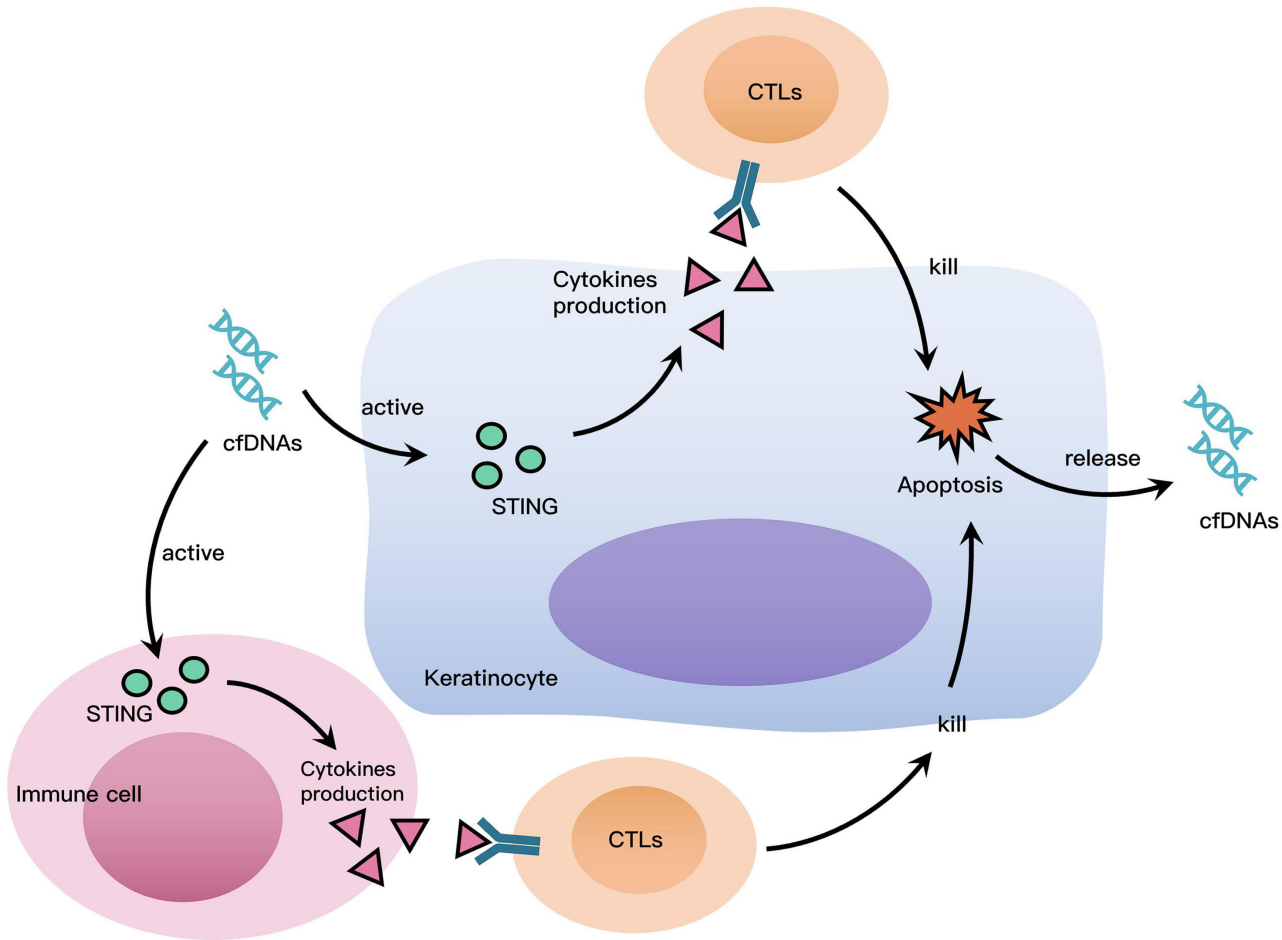
Roles cfDNA Plays in OLP. cfDNA in OLP is recognized by DNA sensors in immune cells and keratinocytes, leading to the activation of STING and production of pro-inflammatory cytokines associated with activated T lymphocytes. CD8^+^ T lymphocytes induce keratinocytes apoptosis by releasing cytotoxins. Apoptotic and dead cells release cfDNA, which further promotes inflammation in OLP, leading to a positive feedback cycle.

cfDNAs access intracellular DNA sensors to promote inflammation, hence, we investigated the role of STING, which is a widely known as a DNA sensor (20). The results of immunohistochemistry and western blotting demonstrated that STING was highly expressed in OLP tissues, and there was also increased protein levels of phosphoryl-STING in OLP tissues, which suggested that the STING pathway was widely activated in OLP. It is worth noting that STING was mainly expressed in lymphocytes rather than keratinocytes, the different uptake capacity to cfDNA of immune cells and keratinocytes might be responsible for this phenomenon. In THP-1 macrophages stimulated by cfDNA-OLP, the expression of STING and the phosphorylation of STING were both highly increased, while siRNA-STING reversed the activation of STING and the inflammation induced by cfDNA-OLP, suggesting that STING activation is the crucial step in inflammation induced by cfDNA-OLP. Finally, PAMAM-G3 inhibited STING activation and reversed the activation of STING and inflammation caused by cfDNA-OLP. Similarly, to our results, two studies reported that cationic polymers can eliminate cfDNA and inhibit nucleic acid-induced inflammation (36, 37). Based on available evidence, including our findings, we suggest that cfDNA plays a crucial role in the inflammatory process of OLP. We propose the following hypothesis to explain the role of cfDNA in OLP: cfDNA in OLP is recognized by DNA sensors in immune cells and keratinocytes, leading to the activation of STING and production of pro-inflammatory cytokines associated with activated T lymphocytes. CD8^+^ T lymphocytes induce keratinocytes apoptosis by releasing cytotoxins. Apoptotic and dead cells release cfDNA, which further promotes inflammation in OLP, leading to a positive feedback cycle (**Figure 5**).

Although some exciting results were obtained in the present study, there are still unclear issues requiring future investigation. It is required to conduct more researches to systematically conclude the relationship between increased cfDNA and OLP, including evaluating the levels of cfDNA in different OLP subtypes, and the levels of cfDNA in OLP patients who receive treatment or not. Although the concentration and sequence of cfDNA both contributed to the pro-inflammation capability of cfDNA-OLP, the genomic distribution and sequence features of cfDNA from OLP patients were unclear, thus, further studies are needed to identify and characterize the cfDNA in OLP. Furthermore, the pro-inflammation capability of cfDNA-saliva was much higher than that of cfDNA-plasma, so it is important to investigate the different sequences of cfDNA-saliva and cfDNA-plasma, which contributes to revealing the clearance mechanism of cfDNA in OLP. Finally, the therapeutic strategy where PAMAM-G3 removes cfDNA to inhibit inflammation needs to be validated *via* an OLP mice model, and the roles of other DNA sensors, such as TLR9 and AIM2, should also be investigated in OLP.

## Conclusion

In conclusion, our study has demonstrated that the cfDNA level in the saliva and plasma of OLP patients were considerably higher than in healthy individuals, correlating with inflammation in OLP patients. cfDNA-OLP induced a significant inflammatory response by activating STING, and the cationic polymer scavenger PAMAM-G3 abrogated this inflammation. This is the first study to report the role of cfDNA in OLP, which proposes a potential therapeutic strategy for OLP using cationic polymers-mediated cfDNA scavenging to reduce the inflammation in OLP.

## Data Availability Statement

The original contributions presented in the study are included in the article/**Supplementary Material**. Further inquiries can be directed to the corresponding authors.

## Ethics Statement

The studies involving human participants were reviewed and approved by Institutional Ethical Committee of West China Hospital of Stomatology, Sichuan University. The patients/participants provided their written informed consent to participate in this study.

## Author Contributions

Conceptualization: JD and LX. Data curation: JD and QMC. Formal analysis: JD. Funding acquisition: LX and QMC. Investigation: JD, WP, NJ, NL, QC, JC, YS, and LX. Methodology: JD, WP, NJ, NL, QC, JC, YS, LX, and QMC. Project administration: JD and LX. Supervision: LX and QMC. Visualization: JD and LX. Writing – original draft: JD. Writing – review and editing: JD, LX and QMC. All authors contributed to manuscript revision, read, and approved the submitted version.

## Funding

This work was supported by the National Natural Science Foundation of China (No. 81730030, 82071137, 81991500, 81991502, U19A2005) and the CAMS Innovation Fund for Medical Sciences (CIFMS) (2019-12M-5-004 and 2020-I2M-C&T-A-023).

## Conflict of Interest

The authors declare that the research was conducted in the absence of any commercial or financial relationships that could be construed as a potential conflict of interest.

## Publisher’s Note

All claims expressed in this article are solely those of the authors and do not necessarily represent those of their affiliated organizations, or those of the publisher, the editors and the reviewers. Any product that may be evaluated in this article, or claim that may be made by its manufacturer, is not guaranteed or endorsed by the publisher.
