# Supplementary material for: Cell-Free DNA Promotes Inflammation in Patients With Oral Lichen Planus via the STING Pathway
Source: Front Immunol. 2022 Apr 14;13:838109. doi: 10.3389/fimmu.2022.838109 (PMC9049180; doi:10.3389/fimmu.2022.838109)
Supplement: Supplementary file 1 [file DataSheet_1.docx]

Supplementary Material

**Supplemental Tables**

**SUPPLEMENTAL TABLE 1** | Primer sequences involved in this study.

| Primer name | Forward(5’-3’) | Reverse(5’-3’) |
| --- | --- | --- |
| IFN-α | GTCTTCACACTCCTGGCACA | GCTTGAGCCTTCTGGATCTG |
| il-6 | CATGAAAATGAAGAGTCCGCAT | GAATGTGAACCATGTTGAGCTT |
| IL-10 | GGAAGCTTTTCTACTCAGCAGA | CACAGATTTTGGCTAGCTCATC |
| NF-κB | TATTTGAAACACTGGAAGCACG | CCGGAAGAAAAGCTGTAAACAT |
| TLR-4 | GACTGGGTAAGGAATGAGCTAG | ACCTTTCGGCTTTTATGGAAAC |
| TNF-α | TGGCGTGGAGCTGAGAGATAACC | CGATGCGGCTGATGGTGTGG |
| STING | GCGGCTGTATATTCTCCTCCCATTG | TGCTGTTGCTGTAAACCCGATCC |

**SUPPLEMENTAL TABLE 2** | siRNA sequences involved in this study.

| Catalogue Number | siRNA Name | Target Sequences |
| --- | --- | --- |
| stB0017344A | genOFFTM st-h-sting_001 | GATCGGGTTTACAGCAACA |
| stB0017344B | genOFFTM st-h-sting_002 | CTGGCATGGTCATATTACA |
| stB0017344C | genOFFTM st-h-sting_003 | GGATTCGAACTTACAATCA |


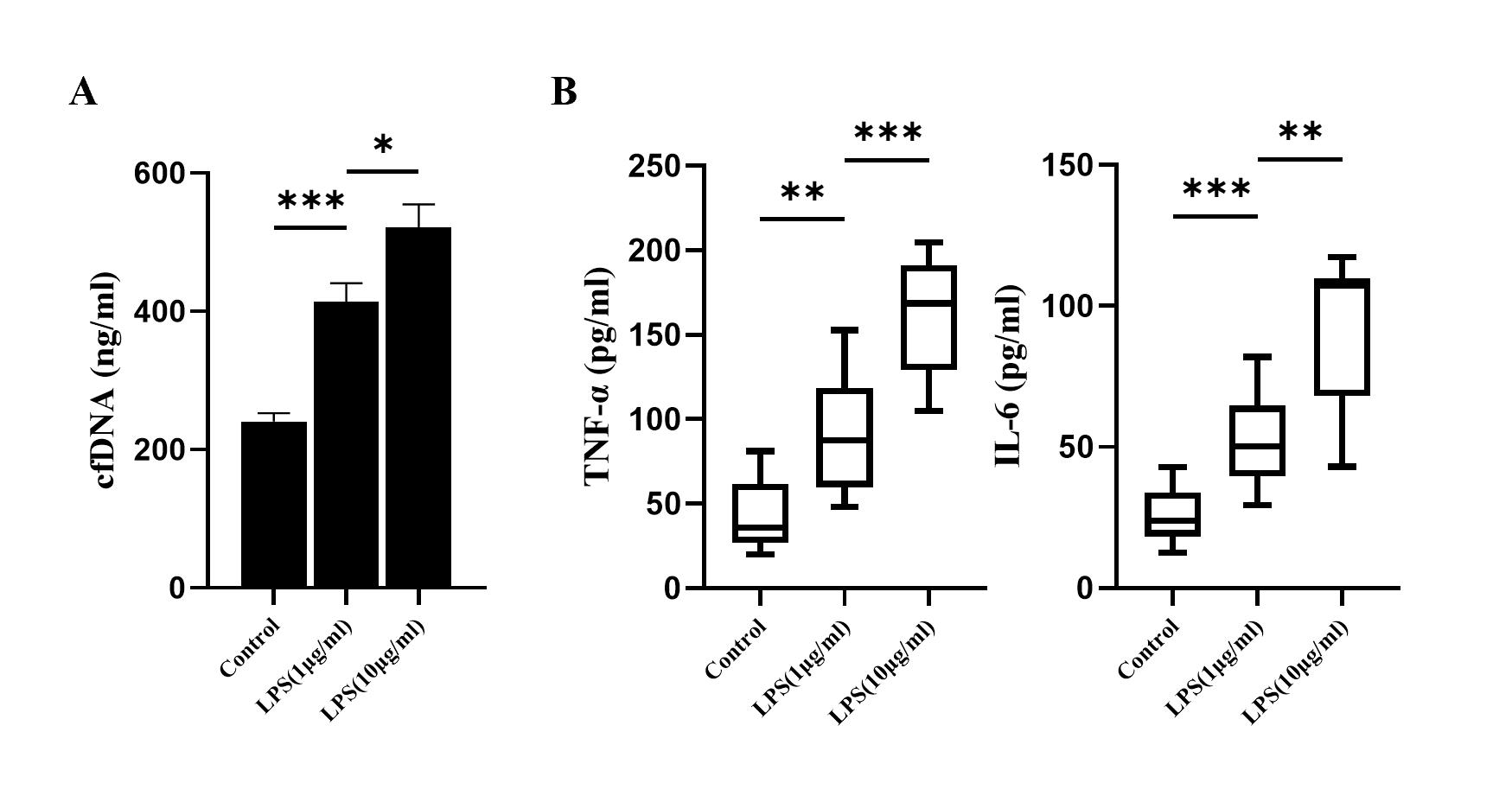
**Supplemental Figure legends**

**SUPPLEMENTAL FIGURE 1** | The levels of cfDNA, TNF-α and IL-6 were significantly increased in supernatant of HOKs treated with LPS. **(A)** The concentrations of cfDNA in supernatant of HOKs treated with LPS were measured by Quant-iT™ PicoGreen™ dsDNA Assay Kit. **(B)** The levels of TNF-α and IL-6 in the supernatants of HOKs were measured using ELISA kits. The data are mean±SEM (*p<0.05, **p<0.01, ***p<0.001).


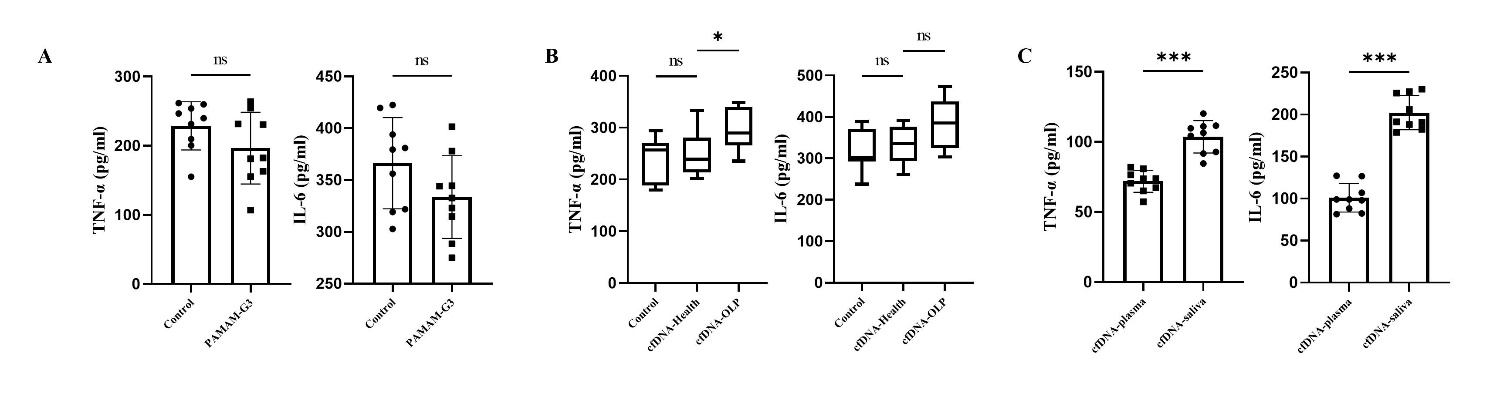


**SUPPLEMENTAL FIGURE 2** | **(A)** Incubated with 25 mg/mL of PAMAM-G3 for 24 h, the levels of TNF-α and IL-6 in the supernatants of THP-1 macrophages were measured using ELISA kits. **(B)** Treated with cfDNA-OLP and cfDNA-Health extracted from plasma for 24 h, the levels of TNF-α and IL-6 in the supernatants of THP-1 macrophages were assessed by ELISA. **(C)** Treated with cfDNA-plasma and cfDNA-saliva from OLP patients for 24 h, the levels of TNF-α and IL-6 in the supernatants of THP-1 macrophages were assessed by ELISA. The data are mean±SEM (*p<0.05, **p<0.01, ***p<0.001). ns, not significant.
